# Understanding the link between the actin cytoskeleton and prion biology

**DOI:** 10.1371/journal.ppat.1011228

**Published:** 2023-03-30

**Authors:** Jane E. Dorweiler, Anita L. Manogaran

**Affiliations:** Department of Biological Sciences, Marquette University, Milwaukee, Wisconsin, United States of America; National Institutes of Health, UNITED STATES

## Introduction

Prion diseases are a family of fatal proteinopathies that are associated with progressive neurodegeneration, such as Creutzfeldt–Jakob disease, bovine spongiform encephalitis, and chronic wasting disease. These diseases are associated with an infectious protein agent, called a prion, rather than bacteria or virus. In mammals, prions are composed of misfolded Prp protein (Prp^Sc^) aggregated into highly organized beta-sheet structures called amyloids. Interestingly, the infectious nature of these amyloids means that prions can convert normal Prp protein into an aggregated form, resulting in a self-perpetuating agent. The ability to propagate the prion is dependent upon the fragmentation of large aggregates into smaller transmissible particles (reviewed in [1]). Animal and cell culture models have provided us with important insight into how prions can cross species barriers, exist in prion conformational states (also called strains), and mechanisms of toxicity. Yet, the molecular underpinnings of how newly formed prion aggregates are managed and transmitted by cells are still unclear.

Similarities between mammalian prions and fungal prions have allowed us to delve into the mechanisms of prion formation and transmission using the basic cellular system of yeast. In 1994, Reed Wickner discovered that 2 yeast proteins, Sup35 and Ure2, could form prions [2]. The next 25 years brought forth exciting findings including that yeast prions behave similarly to mammalian prions, the role of molecular chaperones in prion propagation, and importantly that protein aggregates are the causal form of the infectious prion agent, providing solid proof for the “protein only” hypothesis [3–5].

## The Sup35 prion called [*PSI*^+^]

Since 1994, there have been a diverse array of yeast proteins identified that undergo a structural conversion that is epigenetically heritable through many rounds of cell division, defining them as prions (reviewed in [5,6]). Some of the most recently identified prions are not amyloid in nature [7–9], whereas others, especially the earliest identified yeast prions, are amyloids. [*PSI*^+^] was the first such prion identified in yeast [10] and continues to be among the most studied.

The spontaneous formation of [*PSI*^+^] is rare (1 in 10 million cells), thus making prion formation extremely difficult to study in the lab. However, formation frequencies can be dramatically increased by transiently overexpressing the Sup35 protein in the presence of another prion or overexpression of proteins with glutamine rich domains [11,12]. While overexpression of Sup35 increases the chance a small portion of proteins misfold [2,13], the need for the presence of a preexisting prion is thought to either provide a template for protein aggregation or sequester prion-inhibiting factors away from Sup35 allowing for protein misfolding and aggregation [11,14,15]. Fusion of Sup35 or the N-terminal glutamine/asparagine rich domain (called the prion domain or PrD) to GFP also allows for real-time tracking of prion formation through the presence of microscopically visible puncta. Visible puncta are considered hallmarks of prion formation because cells containing these puncta give rise to future generations that propagate the prion [16,17]. However, the visible puncta are likely not the infectious agent. Visible puncta are asymmetrically retained in mother cells, and non-visible particles (or propagons) are transmitted to daughter cells [18]. The propagation of [*PSI*^+^] from mother to daughter to granddaughter and beyond is dependent upon the generation and transmission of these propagons. The inheritance of propagons through generations is dependent upon the Hsp104 molecular chaperone, which plays an essential role in fragmenting these prion aggregates into smaller transmissible particles [19–21].

## Prion formation and endocytic coat proteins associated with the actin patch

A number of small-scale and large-scale deletion screens have identified genes required for [*PSI*^+^] formation. Several genes identified encode proteins that coordinate actin polymerization at sites of endocytosis and the developing bud, called actin patches. Actin patches are dynamic branched actin structures that require the recruitment of initiation factors near the cell surface. These initiation proteins, commonly called endocytic coat proteins, such as Sla1, Sla2, and Ent1, assemble near the membrane at endocytic sites to coordinate actin assembly and membrane invagination. Interestingly, many of these endocytic coat proteins have prion-like domains (PrLD), which are glutamine and asparagine-rich [22]. Proteins containing PrLDs, including Sup35, have been shown to promote phase separation [23,24]. Recent work has shown that a PrLD containing endocytic coat protein, Ede1, may undergo liquid–liquid phase separation to form condensates that initiate the endocytosis process [25]. Furthermore, other coat proteins such as Sla1 and Ent1, utilize these PrLDs to form rich condensates near sites of endocytosis [26], which possibly mediate membrane invagination.

**Fig 1:**
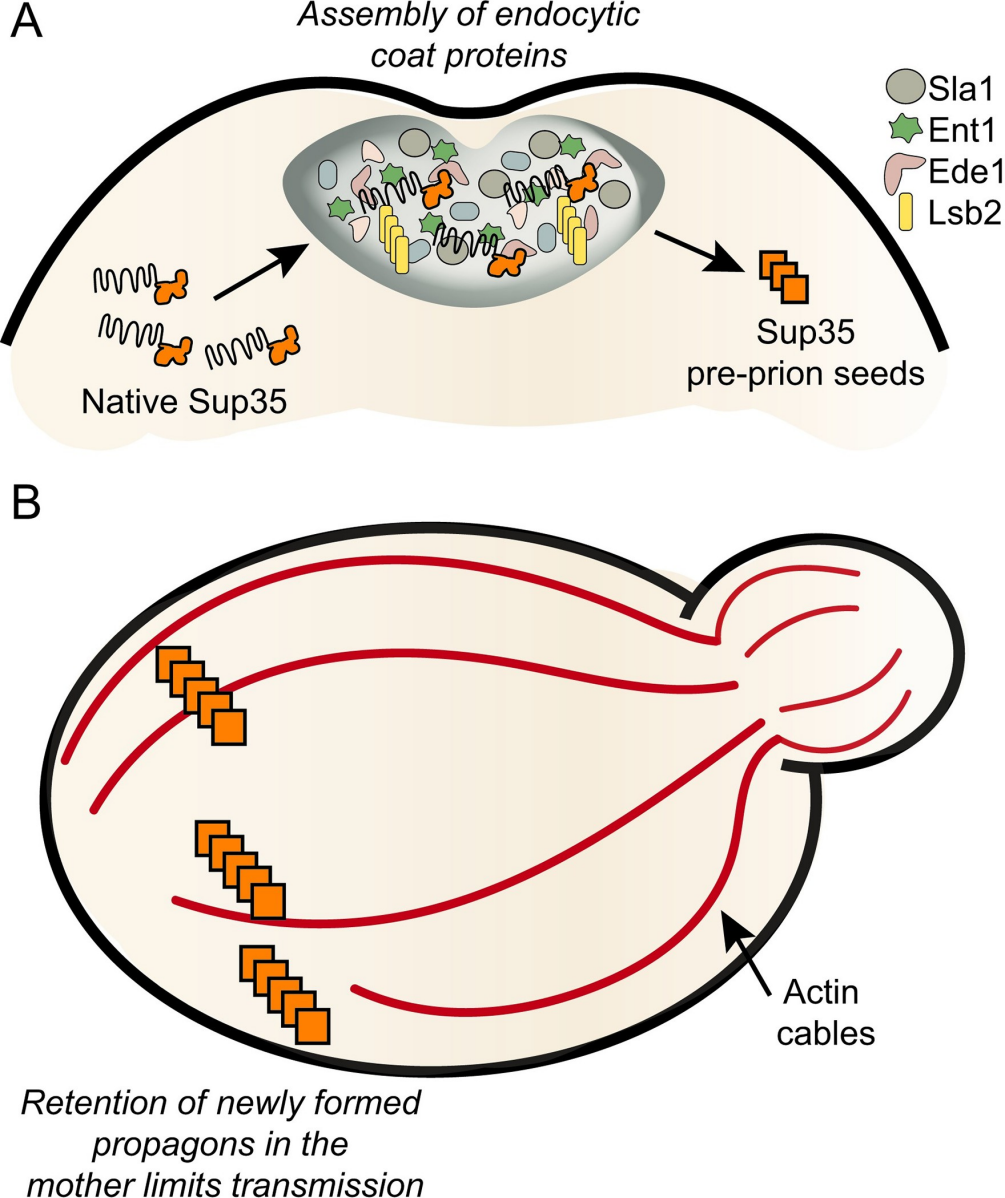
Model of actin in prion formation. (A) PrLD containing endocytic coat proteins (such as Sla1, Ede2, and Ent1, shown as shapes) are suggested to form condensates that mediate the initiation of endocytosis. In addition, the Lsb2 endocytic coat protein forms metastable prions (shown in yellow), although it is unclear whether these metastable prions are associated with the endocytic condensate. The presence of condensates or metastable prions may recruit the prion domain of Sup35 (orange protein with prion domain shown as black line) to nucleate the formation of pre-prion seeds. (B) Actin cable networks (red) have been shown to play a role in the retention of unhealthy organelles or damaged proteins in the mother cell. Recent work has shown that the disruption of actin cables is associated with increased [*PSI*^*+*^] induction frequencies, which may facilitate enhanced transmission of propagons. Therefore, the implications of this study suggest that normal actin cables may play an important role in restricting propagon transmission to daughter cells.

As previously discussed, overexpression of Sup35 or its prion domain fused to GFP results in the formation of microscopically visible puncta and the formation of [*PSI*^+^] [27]. Induced [*PSI*^+^] formation in strains containing deletions of endocytic coat proteins, such as *sla1Δ*, *sla2Δ*, *las17Δ*, *and pan1Δ*, resulted in reduced number of cells with puncta as well as decreased prion formation in cell populations [16,28,29]. Conversely, overexpression of fluorescently tagged Sup35 in the presence of the overexpressed glutamine-rich endocytic protein, Lsb2-GFP, not only forms puncta that transiently co-localize with Sup35, but also leads to the induction of [*PSI*^*+*^], possibly through the metastable formation of a Lsb2 prion [11,30]. Interestingly, the metastable Lsb2 prion can also be induced by heat [31], an environmental stress known to recruit PrLD proteins into stress granules [32]. However, it is important to note that the disruption of actin patches alone, through the use of actin point mutants, retained the ability to form puncta and prions [33], indicating that actin patch coat proteins, but not actin organization, underlie this formation process. The presence of PrLD domains in endocytic coat proteins suggest that the formation of condensates or metastable prions may recruit and convert Sup35 proteins, allowing for nucleation of misfolded Sup35 into pre-prion seeds (Fig 1A). While more research is needed to test this model, it is possible that these Sup35 seeds could be the precursor that then allows for further recruitment and conversion of native Sup35 into the prion form.

## Actin cables and spatial retention of newly formed propagons

In yeast, actin is involved in the formation of several structures including actin patches and actin cables. While actin patches orchestrate membrane changes, such as endocytosis and budding, actin cables are long filamentous assemblies used for polarized transport of cargo and organelles throughout the cell, a function normally reserved for microtubules in metazoans [34,35]. Actin cables also play an important role in spatial retention of unhealthy organelles and damaged proteins to mother cells during cell division. For example, healthy mitochondria tend to segregate to daughter cells while lower functioning oxidized mitochondria are spatially retained in the mother cell [36]. However, the ability to retain unhealthy mitochondria appears to decline with age through reduced stability of actin cables [37]. Damaged proteins and heat-induced aggregates are also spatially retained in mother cells through an Hsp104 and actin-dependent mechanism [38,39]. Recent evidence has suggested that the transmission of newly formed prion propagons follow a similar spatial retention mechanism. [*PSI*^*+*^] formation frequencies are increased when actin cables are disrupted, using genetic or pharmacological manipulation, thereby enhancing the transmission of non-visible propagons [33]. These data suggest that actin cables may play an important role in retaining new propagons in mother cells to limit transmission (Fig 1B).

## Future directions

The study of yeast prions has uncovered mechanisms that play an important role in the prion formation process, specifically factors that coordinate and manage cytoskeletal networks. Based on the yeast system, it is possible that biomolecular condensates or metastable prions may seed prion formation in mammals, yet these mechanisms have been poorly explored in vivo. However, there is in vitro evidence that suggests biomolecular condensates may play a role in seeding of amyloid. Condensates can seed the aggregation of purified human α-synuclein protein, which is associated with Parkinson’s disease (reviewed in [40]). Interestingly in the presence of purified α-synuclein, the human Prp protein can also form biomolecular condensates, which can facilitate Prp’s conversion to amyloid [41]. Further investigation will be required to determine whether these types of mechanisms underlie Prp prion formation.

The yeast system also suggests that actin cable networks play an important role in retaining prion propagons as a potential mechanism to limit transmission. However, whether these mechanisms exist in mammalian systems is unclear. One suggested way that prion particles can be transmitted within the brain is through tunneling nanotubes (TNT). These thin intercellular conduits contain actin filaments that allow the transfer of proteins and organelles, as well as viruses, between cells. TNTs have been shown to also mediate the transmission of Prp^Sc^ from infected cells to non-infected cells [42], suggesting that these actin-based structures may explain how prions are spread throughout the brain. However, it is important to note that aggregated prion protein, Prp^Sc^, is also associated with actin filament destabilization [43], and may influence the integrity of actin networks within TNTs. More work is still required to clearly understand whether transmission of prion particles through TNTs are facilitated by actin networks, or occur as a byproduct of failed spatial retention of prion particles due to impaired actin networks.

## References

[1] Scheckel C, Aguzzi A. Prions, prionoids and protein misfolding disorders. Nat Rev Genet. 2018;19(7):405–418. doi: 10.1038/s41576-018-0011-4 29713012

[2] Wickner RB. [URE3] as an altered URE2 protein: evidence for a prion analog in Saccharomyces cerevisiae. Science. 1994;264(5158):566–569. doi: 10.1126/science.7909170 7909170

[3] King CY, Diaz-Avalos R. Protein-only transmission of three yeast prion strains. Nature. 2004;428(6980):319–323. doi: 10.1038/nature02391 15029195

[4] Tanaka M, Chien P, Naber N, Cooke R, Weissman JS. Conformational variations in an infectious protein determine prion strain differences. Nature. 2004;428(6980):323–328. doi: 10.1038/nature02392 15029196

[5] Liebman SW, Chernoff YO. Prions in yeast. Genetics. 2012;191(4):1041–1072. doi: 10.1534/genetics.111.137760 22879407 PMC3415993

[6] Kushnirov VV, Dergalev AA, Alieva MK, Alexandrov AI. Structural Bases of Prion Variation in Yeast. Int J Mol Sci. 2022;23(10). doi: 10.3390/ijms23105738 35628548 PMC9147965

[7] Chakrabortee S, Byers JS, Jones S, Garcia DM, Bhullar B, Chang A, et al. Intrinsically Disordered Proteins Drive Emergence and Inheritance of Biological Traits. Cell. 2016;167(2):369–381 e12. doi: 10.1016/j.cell.2016.09.017 27693355 PMC5066306

[8] Brown JC, Lindquist S. A heritable switch in carbon source utilization driven by an unusual yeast prion. Genes Dev. 2009;23(19):2320–2332. doi: 10.1101/gad.1839109 19797769 PMC2758746

[9] Chakravarty AK, Smejkal T, Itakura AK, Garcia DM, Jarosz DF. A Non-amyloid Prion Particle that Activates a Heritable Gene Expression Program. Mol Cell. 2020;77(2):251–65 e9. doi: 10.1016/j.molcel.2019.10.028 31757755 PMC6980676

[10] Cox BS. [*PSI*], a cytoplasmic suppressor of super-suppressors in yeast. Heredity. 1965;20.

[11] Derkatch IL, Bradley ME, Hong JY, Liebman SW. Prions affect the appearance of other prions: the story of [PIN(+)]. Cell. 2001;106(2):171–182. doi: 10.1016/s0092-8674(01)00427-5 11511345

[12] Osherovich LZ, Weissman JS. Multiple Gln/Asn-rich prion domains confer susceptibility to induction of the yeast [PSI(+)] prion. Cell. 2001;106(2):183–194. doi: 10.1016/s0092-8674(01)00440-8 11511346

[13] Derkatch IL, Chernoff YO, Kushnirov VV, Inge-Vechtomov SG, Liebman SW. Genesis and variability of [PSI] prion factors in Saccharomyces cerevisiae. Genetics. 1996;144(4):1375–1386. doi: 10.1093/genetics/144.4.1375 8978027 PMC1207691

[14] Keefer KM, Stein KC, True HL. Heterologous prion-forming proteins interact to cross-seed aggregation in Saccharomyces cerevisiae. Sci Rep. 2017;7(1):5853. doi: 10.1038/s41598-017-05829-5 28724957 PMC5517628

[15] Villali J, Dark J, Brechtel TM, Pei F, Sindi SS, Serio TR. Nucleation seed size determines amyloid clearance and establishes a barrier to prion appearance in yeast. Nat Struct Mol Biol. 2020;27(6):540–549. doi: 10.1038/s41594-020-0416-6 32367069 PMC7293557

[16] Ganusova EE, Ozolins LN, Bhagat S, Newnam GP, Wegrzyn RD, Sherman MY, et al. Modulation of prion formation, aggregation, and toxicity by the actin cytoskeleton in yeast. Mol Cell Biol. 2006;26(2):617–629. doi: 10.1128/MCB.26.2.617-629.2006 16382152 PMC1346895

[17] Sharma J, Wisniewski BT, Paulson E, Obaoye JO, Merrill SJ, Manogaran AL. De novo [PSI +] prion formation involves multiple pathways to form infectious oligomers. Sci Rep. 2017;7(1):76. doi: 10.1038/s41598-017-00135-6 28250435 PMC5427932

[18] Mathur V, Hong JY, Liebman SW. Ssa1 Overexpression and [PIN(+)] Variants Cure [PSI(+)] by Dilution of Aggregates. J Mol Biol. 2009;390(2):155–167. doi: 10.1016/j.jmb.2009.04.063 19422835 PMC2738641

[19] Chernoff YO, Lindquist SL, Ono B, Inge-Vechtomov SG, Liebman SW. Role of the chaperone protein Hsp104 in propagation of the yeast prion-like factor [psi+]. Science. 1995;268(5212):880–884. doi: 10.1126/science.7754373 7754373

[20] Shorter J, Lindquist S. Hsp104 catalyzes formation and elimination of self-replicating Sup35 prion conformers. Science. 2004;304(5678):1793–1797. doi: 10.1126/science.1098007 15155912

[21] Satpute-Krishnan P, Langseth SX, Serio TR. Hsp104-dependent remodeling of prion complexes mediates protein-only inheritance. PLoS Biol. 2007;5(2):e24. doi: 10.1371/journal.pbio.0050024 17253904 PMC1779812

[22] Malinovska L, Kroschwald S, Alberti S. Protein disorder, prion propensities, and self-organizing macromolecular collectives. Biochim Biophys Acta. 2013;1834(5):918–931. doi: 10.1016/j.bbapap.2013.01.003 23328411

[23] Franzmann TM, Alberti S. Prion-like low-complexity sequences: Key regulators of protein solubility and phase behavior. J Biol Chem. 2019;294(18):7128–7136. doi: 10.1074/jbc.TM118.001190 29921587 PMC6509491

[24] Franzmann TM, Jahnel M, Pozniakovsky A, Mahamid J, Holehouse AS, Nuske E, et al. Phase separation of a yeast prion protein promotes cellular fitness. Science. 2018;359 (6371). doi: 10.1126/science.aao5654 29301985

[25] Kozak M, Kaksonen M. Condensation of Ede1 promotes the initiation of endocytosis. Elife. 2022:11. doi: 10.7554/eLife.72865 35412456 PMC9064294

[26] Bergeron-Sandoval LP, Kumar S, Heris HK, Chang CLA, Cornell CE, Keller SL, et al. Endocytic proteins with prion-like domains form viscoelastic condensates that enable membrane remodeling. Proc Natl Acad Sci U S A. 2021;118 (50). doi: 10.1073/pnas.2113789118 34887356 PMC8685726

[27] Derkatch IL, Bradley ME, Zhou P, Chernoff YO, Liebman SW. Genetic and environmental factors affecting the de novo appearance of the [PSI+] prion in Saccharomyces cerevisiae. Genetics. 1997;147(2):507–519. doi: 10.1093/genetics/147.2.507 9335589 PMC1208174

[28] Manogaran AL, Hong JY, Hufana J, Tyedmers J, Lindquist S, Liebman SW. Prion formation and polyglutamine aggregation are controlled by two classes of genes. PLoS Genet. 2011;7(5):e1001386. doi: 10.1371/journal.pgen.1001386 21625618 PMC3098188

[29] Speldewinde SH, Doronina VA, Tuite MF, Grant CM. Disrupting the cortical actin cytoskeleton points to two distinct mechanisms of yeast [PSI+] prion formation. PLoS Genet. 2017;13(4):e1006708. doi: 10.1371/journal.pgen.1006708 28369054 PMC5393896

[30] Chernova TA, Romanyuk AV, Karpova TS, Shanks JR, Ali M, Moffatt N, et al. Prion induction by the short-lived, stress-induced protein Lsb2 is regulated by ubiquitination and association with the actin cytoskeleton. Mol Cell. 2011;43(2):242–252. doi: 10.1016/j.molcel.2011.07.001 21777813 PMC3151368

[31] Chernova TA, Kiktev DA, Romanyuk AV, Shanks JR, Laur O, Ali M, et al. Yeast Short-Lived Actin-Associated Protein Forms a Metastable Prion in Response to Thermal Stress. Cell Rep. 2017;18(3):751–761. doi: 10.1016/j.celrep.2016.12.082 28099852 PMC5267347

[32] Boncella AE, Shattuck JE, Cascarina SM, Paul KR, Baer MH, Fomicheva A, et al. Composition-based prediction and rational manipulation of prion-like domain recruitment to stress granules. Proc Natl Acad Sci U S A. 2020;117(11):5826–5835. doi: 10.1073/pnas.1912723117 32127480 PMC7084078

[33] Dorweiler JE, Lyke DR, Lemoine NP, Guereca S, Buchholz HE, Legan ER, et al. Implications of the Actin Cytoskeleton on the Multi-Step Process of [PSI(+)] Prion Formation. Viruses. 2022;14(7). doi: 10.3390/v14071581 35891561 PMC9321047

[34] Pruyne D, Legesse-Miller A, Gao L, Dong Y, Bretscher A. Mechanisms of polarized growth and organelle segregation in yeast. Annu Rev Cell Dev Biol. 2004;20:559–591. doi: 10.1146/annurev.cellbio.20.010403.103108 15473852

[35] Pollard TD, Cooper JA. Actin, a central player in cell shape and movement. Science. 2009;326(5957):1208–1212. doi: 10.1126/science.1175862 19965462 PMC3677050

[36] Higuchi R, Vevea JD, Swayne TC, Chojnowski R, Hill V, Boldogh IR, et al. Actin dynamics affect mitochondrial quality control and aging in budding yeast. Curr Biol. 2013;23(23):2417–2422. doi: 10.1016/j.cub.2013.10.022 24268413 PMC3932488

[37] Sing CN, Garcia EJ, Lipkin TG, Huckaba TM, Tsang CA, Coughlin AC, et al. Identification of a modulator of the actin cytoskeleton, mitochondria, nutrient metabolism and lifespan in yeast. Nat Commun. 2022;13(1):2706. doi: 10.1038/s41467-022-30045-9 35577788 PMC9110415

[38] Hill SM, Hanzen S, Nystrom T. Restricted access: spatial sequestration of damaged proteins during stress and aging. EMBO Rep. 2017;18(3):377–391. doi: 10.15252/embr.201643458 28193623 PMC5331209

[39] Tessarz P, Schwarz M, Mogk A, Bukau B. The yeast AAA+ chaperone Hsp104 is part of a network that links the actin cytoskeleton with the inheritance of damaged proteins. Mol Cell Biol. 2009;29(13):3738–3745. doi: 10.1128/MCB.00201-09 19398583 PMC2698747

[40] Mukherjee S, Sakunthala A, Gadhe L, Poudyal M, Sawner AS, Kadu P, et al. Liquid-liquid Phase Separation of alpha-Synuclein: A New Mechanistic Insight for alpha-Synuclein Aggregation Associated with Parkinson’s Disease Pathogenesis. J Mol Biol. 2022;167713. 35787838 10.1016/j.jmb.2022.167713

[41] Agarwal A, Arora L, Rai SK, Avni A, Mukhopadhyay S. Spatiotemporal modulations in heterotypic condensates of prion and alpha-synuclein control phase transitions and amyloid conversion. Nat Commun. 2022;13(1):1154. 35241680 10.1038/s41467-022-28797-5 PMC8894376

[42] Gousset K, Schiff E, Langevin C, Marijanovic Z, Caputo A, Browman DT, et al. Prions hijack tunnelling nanotubes for intercellular spread. Nat Cell Biol. 2009;11(3):328–336. doi: 10.1038/ncb1841 19198598

[43] Fang C, Wu B, Le NTT, Imberdis T, Mercer RCC, Harris DA. Prions activate a p38 MAPK synaptotoxic signaling pathway. PLoS Pathog. 2018;14(9):e1007283. doi: 10.1371/journal.ppat.1007283 30235355 PMC6147624

